# Challenges and Gaps in the Diagnosis of Personality Disorders in Older Adults: A Review of Current Practices in a UK Mental Health Trust

**DOI:** 10.1192/bjo.2024.461

**Published:** 2024-08-01

**Authors:** Christina Barmpagianni, Jemma Arrow, Neelima Reddi, Tamsin Brownell, Ramin Nilforooshan

**Affiliations:** Surrey and Borders Partnership NHS Foundation Trust, Surrey, United Kingdom

## Abstract

**Aims:**

Personality disorders (PDs) involve persistent deviations from societal norms causing distress, particularly in older UK adults. ICD–11 distinguishes general personality dysfunction from traits. Despite a low reported prevalence among the elderly, underdiagnosis and undertreatment are concerns, suggesting higher actual prevalence. PD presentations in older adults differ, with increasing prevalence noted. Existing research lacks large-scale, population-based studies, longitudinal perspectives, and diagnostic tools sensitive to age-related changes. Overlapping symptoms and delayed diagnosis challenge accurate assessment, while misdiagnosis can lead to repeat hospitalisations.

A UK mental health organisation observed such issues, prompting a diagnostic pathway review and a service evaluation study to identify healthcare professionals' challenges in diagnosing personality disorders in older adults.

**Methods:**

An online survey, conducted from January to March 2023, targeted healthcare professionals in the Trust. It gathered demographic data and focused on professionals' knowledge, and confidence in diagnosing personality disorders, along with limitations and suggestions for improvement. Responses were qualitative, involving community mental health team managers, doctors, healthcare assistants, mental health nurses, occupational therapists, and psychologists. Results, collected in March 2023, aimed to provide detailed insights into professionals' experiences with PDs when treating older adults.

**Results:**

Among 35 surveyed professionals (15 Consultant Psychiatrists, 2 community team managers, 6 nurses, 1 occupational therapist, 2 psychologists, and 9 junior doctors), 75% routinely conducted personality disorder assessments. They lacked specific diagnostic tools, relying on history and ICD–10/DSM–5 criteria. Confidence levels varied, with only 1 reporting high confidence and 37% not confident at all, citing a need for training and structured tools. Challenges in diagnosing older adults were acknowledged by 34 responders, attributing difficulties to comorbidities and ageing. All emphasised the importance of accurate diagnosis for tailored therapy, care, service workload, and healthcare financial implications.

**Conclusion:**

Underdiagnosis and undertreatment of personality disorders in older adults impact their quality of life, posing challenges to healthcare services with financial implications. This local survey and service evaluation study revealed healthcare professionals' lower confidence in diagnosing PDs in older adults, attributed to the complexity of presentation and lack of diagnostic tools. Professionals may underestimate PD prevalence, emphasizing the need for improved education and training. The review calls for validated diagnostic tools tailored to older adults and suggests a need for larger-scale, mixed-methods research to explore factors affecting diagnosis accuracy. It underscores gaps in knowledge and emphasises the importance of understanding and addressing PDs in this population through research, education of professionals, and improved screening.

